# Functional Architecture of Deleterious Genetic Variants in the Genome of a Wrangel Island Mammoth

**DOI:** 10.1093/gbe/evz279

**Published:** 2020-02-07

**Authors:** Erin Fry, Sun K Kim, Sravanthi Chigurapti, Katelyn M Mika, Aakrosh Ratan, Alexander Dammermann, Brian J Mitchell, Webb Miller, Vincent J Lynch

**Affiliations:** 1 Department of Human Genetics, The University of Chicago; 2 Department of Cell and Molecular Biology, Feinberg School of Medicine, Northwestern University; 3 Center for Public Health Genomics, University of Virginia; 4 Max Perutz Labs, Vienna Biocenter (VBC), University of Vienna, Austria; 5 Center for Comparative Genomics and Bioinformatics, Pennsylvania State University; 6 Department of Biological Sciences, University at Buffalo, SUNY

**Keywords:** mammoth, functional evolution, genome evolution

## Abstract

Woolly mammoths were among the most abundant cold-adapted species during the Pleistocene. Their once-large populations went extinct in two waves, an end-Pleistocene extinction of continental populations followed by the mid-Holocene extinction of relict populations on St. Paul Island ∼5,600 years ago and Wrangel Island ∼4,000 years ago. Wrangel Island mammoths experienced an episode of rapid demographic decline coincident with their isolation, leading to a small population, reduced genetic diversity, and the fixation of putatively deleterious alleles, but the functional consequences of these processes are unclear. Here, we show that a Wrangel Island mammoth genome had many putative deleterious mutations that are predicted to cause diverse behavioral and developmental defects. Resurrection and functional characterization of several genes from the Wrangel Island mammoth carrying putatively deleterious substitutions identified both loss and gain of function mutations in genes associated with developmental defects (HYLS1), oligozoospermia and reduced male fertility (NKD1), diabetes (NEUROG3), and the ability to detect floral scents (OR5A1). These data suggest that at least one Wrangel Island mammoth may have suffered adverse consequences from reduced population size and isolation.

## Introduction

The end of the Pleistocene was marked by dramatic environmental change as repeated climate fluctuations gave way to the warmer, more stable Holocene, including the near complete loss of the cold and dry steppe-tundra (also known as the Mammoth steppe) and the extinction of cold-adapted species such as cave bears, cave hyenas, and woolly rhinoceros. Woolly mammoths (*Mammuthus primigenius*) were among the most abundant cold-adapted megafaunal species during the Middle to Late Pleistocene (∼780–12 kyr BP), inhabiting a large swath of steppe-tundra that extended from Western Europe, through Asia and Beringia, into North America. Paleontological and genetic data indicate that their once-large populations experienced at least two demographic declines, the first in the later stages of the Middle Pleistocene ∼285 kyr BP (Palkopoulou et al. 2015) or Eemian interglacial ∼130–116 kyr BP (Palkopoulou et al. 2013) after which populations rebounded, and a final decline around the Pleistocene–Holocene transition (Nyström et al. 2010, 2012; Thomas 2012; Palkopoulou et al. 2013, 2015).

Although mainland woolly mammoths were extinct by ∼10,500 years ago, rising sea levels isolated small populations on St. Paul Island in the Bering sea ∼14,000 years ago and no later than 10,000 years ago on Wrangel Island in the Arctic sea (Vartanyan et al. 2008), both of which survived into the mid-Holocene. Population genetic studies have identified two distinct phases in the extinction of woolly mammoths: An end-Pleistocene decline and extinction of continental populations, particularly in Northern Siberia (Nyström et al. 2010, 2012; Palkopoulou et al. 2013, 2015), followed by the extinction of relict populations on St. Paul Island ∼5,600 years ago (Graham et al. 2016) and Wrangel Island ∼4,000 years ago (Vartanyan et al. 2008; Nyström et al. 2010, 2012; Palkopoulou et al. 2013, 2015) during the mid-Holocene. Although a combination of habitat loss and human hunting likely contributed to the decline and extinction of continental mammoths (Barnosky et al. 2004; Nogués-Bravo et al. 2008; Lorenzen et al. 2011), the synergistic effects of shrinking island area and freshwater scarcity caused by continued sea level rise likely caused the extinction of St. Paul Island mammoths (Graham et al. 2016).

The causes of the extinction of Wrangel Island mammoths are unclear. However, they experienced a period of rapid demographic decline coincident with their isolation, resulting in a small population, reduced genetic diversity, and recurrent breeding among distant relatives, an elevated number of putatively deleterious alleles, and fixation of at least one putatively deleterious mutation in the mitochondrial *ATP6* gene (Nyström et al. 2010, 2012; Thomas 2012; Pečnerová et al. 2016, 2017; Rogers and Slatkin 2017). These data suggest that their extinction was associated with a “mutational meltdown” (Rogers and Slatkin 2017), but the functional consequences of putatively deleterious amino acid substitutions in the Wrangel Island mammoth are unknown. Here, we identify and characterize the functional architecture of genetic variants in the Wrangel Island mammoth genome. We found that putatively damaging substitutions unique to the Wrangel Island mammoth are enriched for numerous deleterious phenotypes, such as reduced male fertility and neurological defects. Functional characterization of several resurrected Wrangel Island mammoth genes indicates that mutations in these genes were indeed deleterious and may have adversely effected development, reproduction, and olfaction.

## Materials and Methods

### Genome Assembly

Details of the sequencing protocol for the Oimyakon and Wrangel Island mammoths can be found in Palkopoulou et al. (2015) and for the Asian elephants, M25, and M4 in Lynch et al. (2015). Briefly, sequences were aligned to the African Savannah elephant (*Loxodonta africana*) reference genome from the (loxAfr3) using the Burrows Wheeler Aligner (Li and Durbin 2010) with default parameters (BWA-SW version 0.5.9-r16). The reads were subsequently realigned around putative indels using the GATK (DePristo et al. 2011) IndelRealigner (version 1.5-21-g979a84a), and putative PCR duplicates were flagged using the MarkDuplicates tool from the Picard suite (version 1.96). Data for the Asian elephants, mammoth M25, and mammoth M4 samples are available from NCBI BioProject collection PRJNA28111 and data for the Oimyakon and Wrangel Island mammoth genomes are available from the European Nucleotide Archive under the accession number ERP008929.

The sequences from the mammoths were treated separately to account for DNA damage in the sequences. Putative adapter sequences were removed and we merged overlapping paired-end reads using available scripts (Kircher 2012). We required an overlap of at least 11 nucleotides between the mates, and only pairs that could be merged were retained for subsequent analyses. The merged reads were aligned to the genome from the African elephant (loxAfr3) using BWA with default parameters, and only mapped reads ≥30 bp were used for single-nucleotide polymorphism calls. The reads were realigned using the GATK IndelRealigner and putative PCR duplicates were flagged using MarkDuplicates, similar to the process described for the modern genomes. We also limited the incorporation of damaged sites into the variant-calling pipeline by hard-masking all sites that would be potentially affected by the characteristic ancient DNA patterns of cytosine deamination in single stranded overhangs. This mask was applied to ten nucleotides on both ends of the merged reads from the ancient samples.

Single-nucleotide variants, that is, positions in the African elephant reference assembly at which we detected a nucleotide different from the reference in at least one of the Asian elephant or mammoth individuals were identified using SAMtools (Li et al. 2009) (version 0.1.19), which was applied with “-C50” to adjust the mapping quality of the reads with multiple mismatches. We did not call differences in regions where the reference base was unknown, and the calls were limited to regions that were covered at least 4 times, and at most 350 times by the sequences in these samples. We then identified homozygous nonsynonymous substitutions unique (private) to each elephant and mammoth genome; these homozygous nonsynonymous substitutions were used in downstream analyses.

The M25 genome is likely a composite of multiple mammoth individuals (Rogers and Slatkin 2017), therefore, we did not include M25 in our functional analyses (described below). However, because we are primarily interested in private nonsynonymous variants within each elephant and mammoth genome, we were able to take advantage of the composite nature of the M25 genome by excluding homozygous nonsynonymous substitutions identified in the three Asian elephants and the three mammoths that were also observed in M25. The rationale for this filtering process is that any homozygous nonsynonymous substitution observed in either the Asian elephants or the three mammoths that is also observed in M25 is not truly a private variant. We thus identified 106 homozygous amino acid substitutions in 99 genes in the Oimyakon mammoth, 162 homozygous amino acid substitutions in 143 genes in the M4 mammoth, and 594 homozygous amino acid substitutions in 525 genes in the Wrangel Island mammoth.

### Functional Annotation

To infer the putative functional consequences of amino acid substitutions in the Wrangel Island mammoth genome, we focused on homozygous derived variants that were not observed in the other mammoth or elephant samples. Our focus on homozygous, derived variants excludes possible deleterious heterozygous variants but reduces the risk of misclassifying amino acid variants that arise from DNA damage. We used PolyPhen-2 to classify amino acid substitutions as “benign,” “possibly damaging,” or “probably damaging” (Adzhubei et al. 2010, 2013) and Enrichr (Chen et al. 2013; Kuleshov et al. 2016) to infer the functional consequences of homozygous “probably damaging” amino acid substitutions in each mammoth and elephant; We note that one Asian elephant is predicted to have a high number of predicted probably damaging amino acid variants, however, with only three individuals it is difficult to know whether this individual is truly an outlier or within the range for species if we had a larger sample size. We then intersected these phenotypes to identify those unique to the Wrangel Island mammoth. We report (unique) enriched mouse knockout phenotypes at an FDR ≤ 0.20. We also used the same approach to identify tissues in which genes with “probably damaging” amino acid substitutions in the Wrangel Island mammoth are enriched.

### Data Availability

Homozygous nonsynonymous substitutions unique (private) in the three extant Asian elephants and mammoths and PolyPhen-2 functional annotations for each variant are available at Galaxy and are included as supplementary materials, Supplementary Material online.

### Selection of Target Genes for Functional Validation

We manually curated each gene with a predicted probably damaging amino acid substitutions in the Wrangel Island mammoth based on literature searches and selected targets for functional validation based on three criteria: 1) the Wrangel Island mammoth specific amino acid substitution must have been classified by PolyPhen-2 as “probably damaging” with a pph2_prob score ≥0.958, 2) prior studies (based on literature reviews) must have identified the molecular function for that gene, and 3) the ability to design straightforward experimental systems in which to test the function of ancestral and derived amino acid variants. Finally, we selected genes for functional validation that had high read coverage to ensure base calls were correct and excluded substitutions at CpG sites. Based on these criteria, we selected HYLS1, NKD1, NEUROG3, and OR5A1 for functional validation.

### HYLS1 Functional Validation

*Xenopus* embryos were acquired by in vitro fertilization using standard protocols (Peter et al. 2001) approved by the Northwestern University Institutional Animal Care and User Committee. Previously validated morpholino oligos (MOs) (GeneTools) were used (Control MO, 5′-CCTCTTACCTCAGTTACAATTTATA-3′; HYLS-1.1, 5′-GAACTGCCTGTCTCGAAGTGACATG-3′; XHYLS-1.2, 5′-GAACTGCCTGTCTCTCAGTGACATG-3′ [Dammermann 2009]). Full length XHYLS1 and the Wrangel mammoth mutant *Xenopus* equivalent XHYLS1-S186L were cloned into pCS2+ and fused with GFP at the N terminus. mRNA of the pCS2 constructs was prepared using in vitro transcription with SP6 (Promega). Morpholinos and mRNA were coinjected into each blastomere at the 2–4 cell stage using a total of 50–75 ng of morpholino and 500 pg to 1 ng mRNA per embryo. Embryos were allowed to develop until stage 28 then fixed with 4% PFA in PBS for 2 h at RT. For antibody staining embryos were blocked for 1 h in PBS with 0.1% Triton and 10% Normal Goat Serum prior to overnight incubation with primary antibody (Acetylated tubulin, Sigma T6793). Fluorescent secondary Abs (Jackson Labs) were incubated overnight after a full day of washing in PBS-0.1 %Triton. After secondary washing, embryos were stained with fluorescently tagged phalloidin to mark the cell boundaries. Imaging was performed on a laser-scanning confocal microscope (A1R; Nikon) using a 60× oil Plan-Apo objective with a 1.4 NA.

### NKD1 Functional Validation

To infer if the A88V substitution had functional affects, the ancestral mammoth (AncYakut, A88) and Wrangel Island (V88) NKD1 genes were synthesized by GeneScript (Piscataway, NJ) using mouse codon usage tables and cloned into the mammalian expression vector pcDNA3.1+C-DYK; we used the most frequently used codon for each amino acid encoded by more than one codon; this generally allows for greater translational efficiency and ensures robust protein expression. Next, we tested their ability to antagonize luciferase expression from the pGL4.49[*luc2P*/TCF-LEF/Hygro] luciferase reporter vector, which drives luciferase expression from a minimal promoter and eight copies of a TCF-LEF response element upon activation of Wnt-signaling. African elephant primary dermal fibroblasts (San Diego Zoo, “Frozen Zoo”) were grown at 37 °C/5% CO_2_ in a culture medium consisting of FGM/MEM (1:1) supplemented with 10% FBS, 1% glutamine, and 1% penstrep. Confluent cells in 96-well plates in 60 µl of Opti-MEM (GIBCO) were transfected with 100 ng of the luciferase reporter plasmid pGL4.49[*luc2P*/TCF-LEF/Hygro], 100 ng of the AncYakut or Wrangel Island mammoth NKD1 expression vector, and 10 ng of pRL-null with 0.1 µl of PLUS reagent (Invitrogen) and 0.3 µl of Lipofectamine LTX (Invitrogen) in 20 µl of Opti-MEM. The cells were incubated in the transfection mixture for 6 h until the transfection media was replaced with culture media and supplemented with the small molecule Wnt-signaling agonist CHIR99021. Forty-eight hours after transfection, Dual Luciferase Reporter Assays (Promega) began with incubating the cells for 15 min in 20 µl of 1× passive lysis buffer. Luciferase and Renilla activity was measured with the Glomax multi+detection system (Promega). We standardized luciferase activity values to Renilla activity values and background activity values by measuring luminescence in wells lacking the NKD1 expression vector.

### NEUROG3 Functional Validation

To determine if the G195E substitution had functional effects, the ancestral mammoth (AncYakut, G195) and Wrangel Island (E195) *NEUROG3* genes were synthesized by GeneScript with mouse codon usage and cloned into the mammalian expression vector pcDNA3.1+C-DYK. Next, we tested their ability to transactivate luciferase expression from the pGL3 luciferase reporter vector containing a minimal promoter and six repeats of the *PAX4* E-box (pGL3 [*luc/6x-PAX4E/minP*]); the *PAX4* E-box has previously been shown to physically bind NEUROG3 and drive luciferase expression in reporter assays (Smith et al. 2004). African Savanah elephant primary dermal fibroblasts (San Diego Zoo, “Frozen Zoo”) were grown at 37 °C/5% CO_2_ in a culture medium consisting of FGM/MEM (1:1) supplemented with 10% FBS, 1% glutamine, and 1% penstrep. Confluent cells in 96-well plates in 60 µl of Opti-MEM (GIBCO) were transfected with 100 ng of the luciferase reporter plasmid 6x-PAX4E_pGL3-Basic, 100 ng of the AncYakut or Wrangel Island mammoth NEUROG3 expression vector, and 10 ng of pRL-null with 0.1 µl of PLUS reagent (Invitrogen) and 0.3 µl of Lipofectamine LTX (Invitrogen) in 20 µl of Opti-MEM. The cells were incubated in the transfection mixture for 6 h until the transfection media was replaced with culture media. Forty-eight hours after transfection, Dual Luciferase Reporter Assays (Promega) began with incubating the cells for 15 min in 20 µl of 1× passive lysis buffer. Luciferase and Renilla activity was measured with the Glomax multi+detection system (Promega). We standardized luciferase activity values to Renilla activity values and background activity values by measuring luminescence in wells lacking the NEUROG3 expression vector.

### OR5A1 Functional Validation

We used the Hana3A odorant receptor assay to determine if the Wrangel Island mammoth OR5A1 S193F substitution had functional effects. This assay system utilizes HEK293 cells stably expressing accessory proteins required for signal transduction by odorant receptors, including RTP1L, RTP2, REEP1, and G_αolf_ (Hana3A cells) (Saito et al. 2004; Zhuang and Matsunami 2008). The ancestral mammoth (AncYakut, S193) and Wrangel Island (F193) *OR5A1* genes were synthesized by GeneScript with human codon usage and into the mammalian expression vector pcDNA3.1+C-DYK. Next, we followed the Hana3A odorant receptor assay protocol to assay their sensitivity to β-ionone (Zhuang and Matsunami 2008) with slight modifications (described next). Hana3A cells were maintained at 37 °C/5% CO_2_ in a culture medium consisting of FGM/MEM (1:1) supplemented with 10% FBS, 1% glutamine, and 1% penstrep. Confluent cells in 96-well plates in 60 µl of Opti-MEM (GIBCO) were transfected with 100 ng of the cAMP responsive CRE-Luc luciferase reporter plasmid, 100 ng of the AncYakut or Wrangel Island mammoth *OR5A1* expression vector, and 10 ng of the SV40-Renilla with 0.1 µl of PLUS reagent (Invitrogen) and 0.3 µl of Lipofectamine LTX (Invitrogen) in 20 µl of Opti-MEM. The cells were incubated in the transfection mixture for 6 h until the transfection media was replaced with culture media. Forty-eight hours after transfection, media was replaced with serum-free CD293 suspension culture medium (GIBCO) supplemented with l-glutamine and increasing concentrations of β-ionone (Sigma). Relative luminescence was assayed 4 h after treatment with β-ionone using the Dual Luciferase Reporter Assays (Promega) as described above. We standardized luciferase activity values to Renilla activity values and background activity values by measuring luminescence in wells lacking the *OR5A1* expression vector.

## Results

To characterize the functional architecture of deleterious variants in mammoth genomes, we used previously available genomes to identify homozygous nonsynonymous substitutions unique (private) in three extant Asian elephants (*Elephas maximus*; Uno, Asha, and Parvathy) and three woolly mammoths—the ∼44,800 year old Oimyakon mammoth (Palkopoulou et al. 2015), the ∼20,000 year old M4 mammoth (Dikov 1988; Gilbert et al. 2007, 2008; Miller et al. 2008; Lynch et al. 2015), and the ∼4,300 year old Wrangel Island mammoth (Palkopoulou et al. 2015). These mammoths span the age from when mammoth populations were large and widespread (Oimyakon), to near the beginning of their final decline (M4), and their last known population (Wrangel Island); Thus, these individuals allow us to compare older variants to variants unique to the last population of mammoths. To reduce potential false positives resulting from damaged sites and other sources of error associated with ancient DNA, we hard-masked all sites that would be potentially affected by the characteristic ancient DNA patterns of cytosine deamination in single stranded overhangs from the variant-calling pipeline. This mask was applied to ten nucleotides on both ends of the merged reads from the ancient samples. The effect of this hard-masking is to reduce the total number of variants called but increase confidence that the called variants are real rather than artifacts of DNA preservation and damage.

**Fig. 1.**
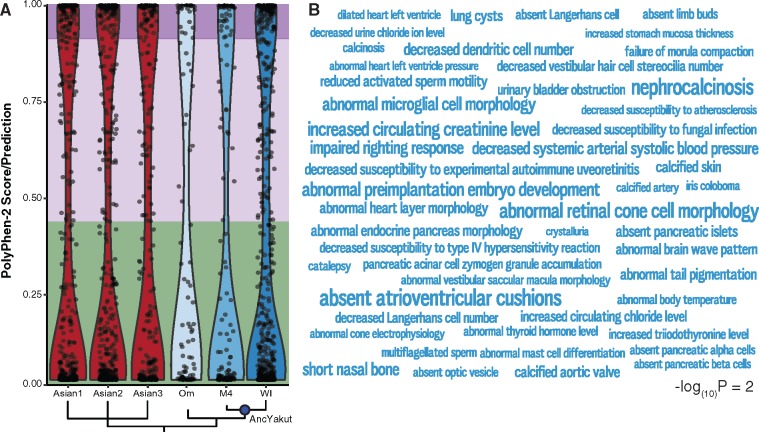
—Functional architecture of deleterious variants in the Wrangel Island mammoth genome. (*A*) Violin plot of PolyPhen-2 scores for derived, homozygous variants in each mammoth and Asian elephant. Phylogenetic relationships are shown at the bottom and the ancestral Yakut (AncYakut) node is indicated with a blue circle. Green, light purple, and dark purple backgrounds indicate variants that are predicted to be benign, possibly damaging, and probably damaging, respectively. (*B*) Word Cloud showing mouse KO phenotypes in which homozygous probably damaging amino acid substitutions in the Wrangel Island mammoth are enriched. In this figure, the size of each phenotype is drawn proportional to the −log_(10)_*P* value of that terms enrichment. Inset scale shows word size for phenotypes with a −log_(10)_*P* value of 2.

We aligned sequencing reads to the genome assembly for the African Savannah elephant (*Loxodonta africana*), resulting in nonredundant average sequence coverage of ∼11-fold for the Oimyakon mammoth genome, ∼20-fold M4 mammoth genome, ∼17-fold for the Wrangel Island genome, and ∼30-fold for each Asian elephant. We identified ∼33 million putative single-nucleotide variants across the 6 genomes, including 106–594 private nonsynonymous substitutions in 106–583 genes. Next, we used PolyPhen-2 (Adzhubei et al. 2010, 2013) to computationally predict the functional impact of each private homozygous amino acid substitution (table 1 and fig. 1*A*) and identified mouse knockout phenotypes and tissues in which these genes were enriched. We identified 115 “probably damaging” amino acid variants in 112 genes in the Wrangel Island mammoth genome. These genes were enriched for 102 mouse knockout phenotypes at an FDR ≤ 0.20 (supplementary table 1, Supplementary Material online) and 63 tissues at an FDR ≤ 0.22 (supplementary table 1, Supplementary Material online) that were not observed as enriched in the deleterious variants from M4 or Oimyakon, nor any of the Asian elephants (fig. 1*B*). Genes with “probably damaging” amino acid variants in the Wrangel Island mammoth genome were enriched for diverse KO phenotypes, including behavioral and neurological defects such as “impaired righting response” (*P *=* *0.008, FDR *q *=* *0.19), “abnormal brain wave pattern” (*P *=* *0.017, FDR *q *=* *0.19), “catalepsy” (*P *=* *0.026, FDR *q *=* *0.19), and “abnormal substantia nigra morphology” (*P *=* *0.038, FDR *q *=* *0.20).

**Table 1:** PolyPhen-2 Classification of Private Amino Acid Variants in Asian Elephants and Woolly Mammoths

Mammoth	Probably Damaging	Possibly Damaging	Benign	Unclassified	Total	Average Coverage
Asian 1	91 (80)	75 (73)	250 (221)	15 (14)	431 (388)	30
Asian 2	130 (124)	75 (72)	295 (259)	22 (21)	522 (476)	30
Asian 3	70 (64)	51 (49)	169 (151)	10 (10)	300 (274)	30
Oimyakon	19 (19)	18 (18)	65 (65)	4 (4)	106 (106)	11
M4	32 (31)	22 (22)	103 (103)	4 (4)	161 (161)	20
Wrangel Island^a^	115 (112)	109 (109)	349 (341)	21 (21)	594 (583)	17

NOTE.—Total number in each category/percent in category. Numbers in parentheses indicate the number of genes with those variants.

^a^Note that the number of private mutations is likely inflated for the Wrangel Island mammoth because only one genome is available.

**Fig. 2:**
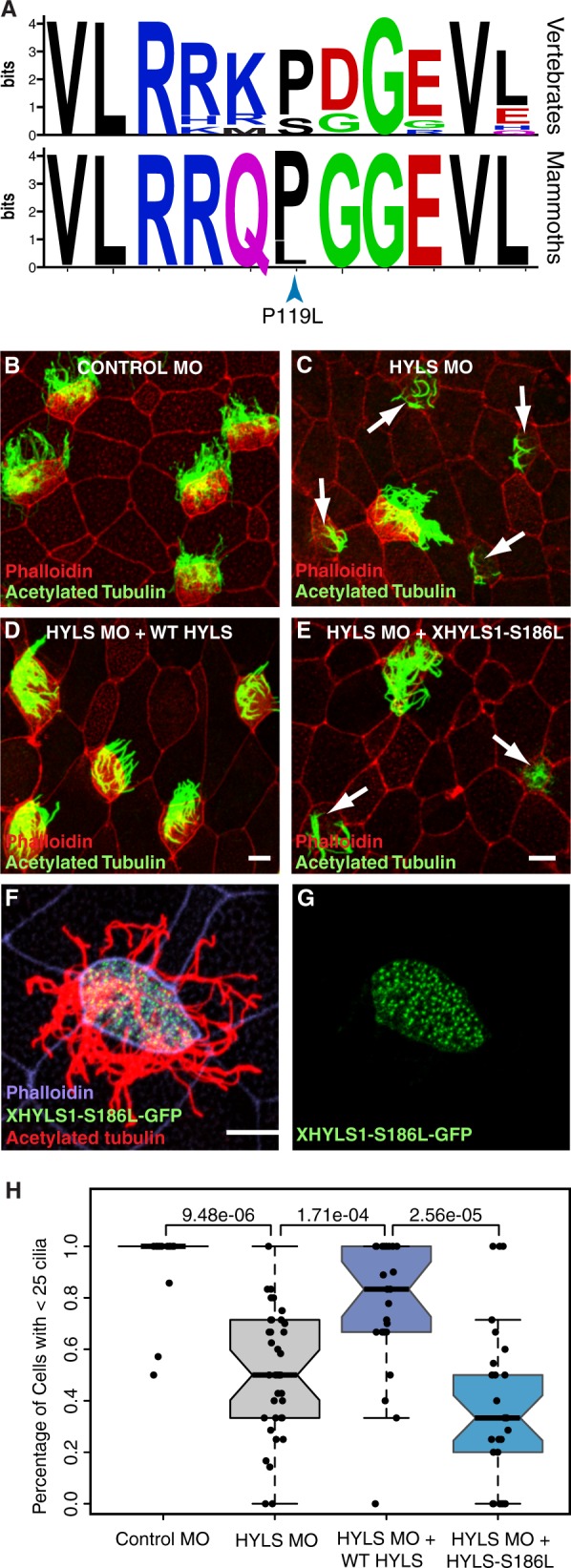
—The Wrangel Island mammoth HYLS1 P119L mutant is dysfunctional. (*A*) Sequence logo showing conservation of HYLS1 around the Wrangel Island mammoth P119L mutation. Upper, logo from 100 vertebrates. Lower, logo from mammoths (Oimyakon, M4, and Wrangel Island). Note that the maximum information (conservation) of an amino acid site is log 2 20 = 4.3 bits. (*B*–*E*) Representative images of *Xenopus* multiciliated cells from embryos injected with Control MO (*B*), HYLS1 MO (*C*), HYLS1 MO and WT XHYLS1 (*D*), and HYLS1 MO and XHYLS1-S186L; note that S186L is the equivalent of the Wrangel Island mammoth genome P119L in *Xenopus* HYLS1 (*E*) stained with acetylated tubulin to mark cilia (green) and phalloidin to mark cell boundaries (red). *Note:* arrows identify cells with defective ciliogenesis (scale bars = 10 μm). (*F*, *G*) Multiciliated cell from a *Xenopus* embryo injected with mRNA encoding XHYLS1-S186L-GFP shows proper localization to the centrioles (green) and proper formation of cilia marked with acetylated tubulin (red) with cell boundaries marked with phalloidin (purple) (scale bar = 10 μm). (*H*) Quantification of ciliogenesis defect scoring cells with >25 cilia (Wilcox test *P* values are shown).

Among the genes with a deleterious variant in the Wrangel Island mammoth genome with behavioral and neurological functions is hydrolethalus syndrome protein 1 (*HYLS1*), a centriolar protein that functions in ciliogenesis (Dammermann et al. 2009). The Wrangel Island mammoth HYLS1 P119L (c.356C>T) mutation occurs in a highly conserved region of the protein, which is invariant for proline or serine across vertebrates and is therefore potentially deleterious (fig. 2*A*). To infer if this mutation had functional consequences, we used a well-characterized *Xenopus* model of ciliogenesis (Dammermann et al. 2009). MOs targeting *Xenopus HYLS1* led to a severe defect in cilia assembly (Wilcox test, *P *=* *9.48 × 10^−6^; fig. 2*C*–*H*), as previously reported (Dammermann et al. 2009). This defect was rescued by addition of MO-resistant wild-type *Xenopus HYLS1* (Wilcox test, *P *=* *1.71 × 10^−4^; fig. 2*D*–*H*), but not a variant incorporating the equivalent P119L mutation into *Xenopus* HYLS1 (S186L) (Wilcox test, *P *=* *2.56 × 10^−5^; fig. 2*E*–*H*). The HYLS1 S186L mutant did, however, appropriately localize to centrioles and did not have any dominant negative effects in the absence of depletion of the endogenous protein (fig. 2*F* and *G*). Mutations in HYLS1 underlie hydrolethalus syndrome (MIM: 236680), a perinatal lethal developmental disorder characterized by severe brain malformation including hydrocephalus and absent midline structures (Mee et al. 2005), as well as Joubert syndrome (MIM: 213300), a milder disorder characterized by defects in the cerebellum and brain stem leading to impaired balance and coordination (Oka et al. 2016), suggesting the HYLS1 P119L mutation may have had adverse developmental consequences.

**Fig. 3:**
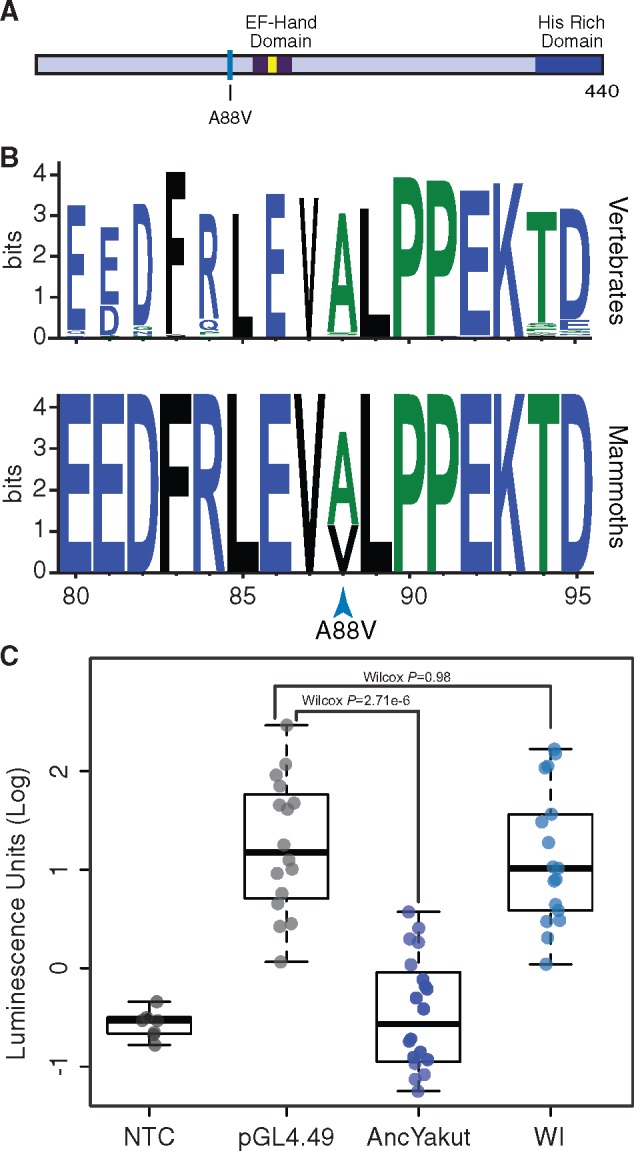
—The Wrangel Island mammoth NKD1 A88V substitution is a loss of function mutation. (*A*) The Wrangel Island mammoth A88V mutation is located near the EF-hand domain (dark blue), the calcium binding motif is shown in yellow. (*B*) Sequence logo showing conservation of NKD1 around the Wrangel Island A88V mutation. Upper, logo from 100 vertebrates. Lower, logo from mammoths (Oimyakon, M4, and Wrangel Island). (*C*) Luciferase expression from the pGL4.49[*luc2P*/TCF-LEF/Hygro] reporter vector in elephant fibroblasts transfected with control vector, AncYakut *NKD1*, or Wrangel Island (WI) Mammoth *NKD1*, and treated with the WNT-signaling agonist CHIR99021. Background luminescence of nontransfected cells (NTC).

Defects in sperm morphology are among the most common consequences of reduced genetic diversity and inbreeding (Asa et al. 2007; Shorter et al. 2017), and several knockout phenotypes only observed in the Wrangel Island genome are related to sperm biology such as “reduced activated sperm motility” (*P *=* *0.011, FDR *q *=* *0.19), “multiflagellated sperm” (*P *=* *0.023, FDR *q *=* *0.19), and “abnormal sperm axoneme morphology” (*P *=* *0.045, FDR *q *=* *0.19); “sperm flagellum” was also the tissue most significantly enriched deleterious variants (*P *=* *0.001, FDR *q *=* *0.22). Among the genes with a “probably damaging” amino acid substitution in the Wrangel Island mammoth associated with sperm defects and male infertility is *Naked cuticle 1* (*NKD1*), which encodes a passive antagonist of the Wnt/TCF-LEF signaling pathway (Van Raay et al. 2007, 2011; Angonin and Van Raay 2013). The Wrangel Island mammoth A88V substitution occurred at a site that is nearly invariant for alanine or serine in diverse vertebrates (fig. 3*A*), suggesting it may have had functional consequences.

To determine if the NKD1 A88V (c.163C>T) substitution had functional effects, we resurrected the Wrangel Island/M4 ancestral (AncYakut, fig. 3*A*) and Wrangel Island *NKD1* genes and tested their ability to antagonize Wnt-signaling in elephant dermal fibroblasts transiently transfected with a luciferase reporter vector containing a minimal promoter and eight copies of a TCF-LEF response element (pGL4.49[*luc2P*/TCF-LEF/Hygro]) and treated with a small molecule agonist of the Wnt-signaling pathway (CHIR99021). The AncYakut NKD1 reduced luminescence to background levels in response to CHIR99021 treatment (Wilcox test, *P *=* *2.71 × 10^−6^). In stark contrast however, the Wrangel Island NKD1 did not affect luciferase expression (Wilcox *P *=* *0.98), indicating that the NKD1 A88V substitution is a loss of function mutation (fig. 3*B*). Transgenic mice with loss of function mutations in NKD1 have dysregulated Wnt/beta-catenin signaling in the testis leading to abnormal seminiferous tubule morphology, small seminiferous tubules, small testis, oligozoospermia, and reduced fertility (Li et al. 2005; Zhang et al. 2007), suggesting this substitution may have affected male fertility.

**Fig. 4:**
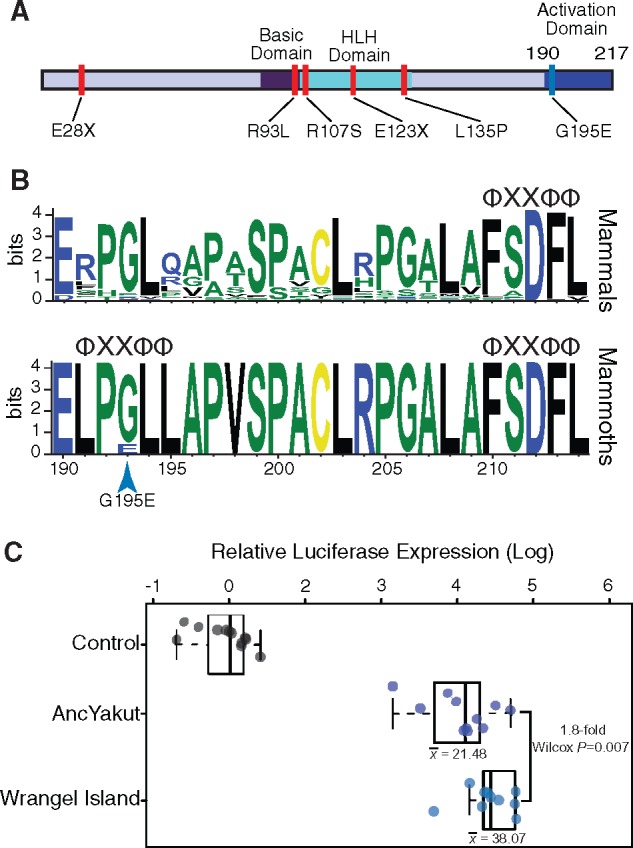
—The Wrangel Island mammoth NEUROG3 is a hypermorph. (*A*) The Wrangel Island mammoth G195E mutation is located in the C-terminal transactivation domain of NEUROG3; location of mutations associated with human disease is shown in red. (*B*) Sequence logo showing conservation of NEUROG3 transactivation domain in mammals (upper) and mammoths (lower; Oimyakon, M4, and Wrangel Island). The location of ΦXXΦΦ cofactor interaction motifs are shown (Φ, any hydrophobic amino acid; X, any amino acid). (*C*) Relative luciferase expression from the pGL3[*luc/6x-PAX4E/minP*] reporter vector in elephant fibroblasts transfected with control vector, AncYakut *NEUROG3*, or Wrangel Island mammoth *NEUROG3*.

Although deleterious Wrangel Island mammoth variants are enriched in diverse KO phenotypes, many are related to the pancreas such as “abnormal endocrine pancreas morphology” (*P *=* *0.011, FDR *q *=* *0.19), “absent pancreatic islets” (*P *=* *0.013, FDR *q *=* *0.19), “pancreatic acinar cell zymogen granule accumulation” (*P *=* *0.019, FDR *q *=* *0.19), “absent pancreatic alpha cells” (*P *=* *0.023, FDR *q *=* *0.19), and “absent pancreatic beta cells” (*P *=* *0.027, FDR *q *=* *0.19). Among the genes with deleterious Wrangel Island variants annotated with “abnormal endocrine pancreas morphology” is *NEUROGENIN 3* (*NEUROG3*), which encodes a basic helix–loop–helix transcription factor that is required for endocrine cell development. The “probably damaging” NEUROG3 G195E (c.584G>A) substitution in the Wrangel Island mammoth NEUROG3 protein occurred at a site that is nearly invariant for glycine across mammals (fig. 4*A* and *B*) within an LXXLL motif in the C-terminal transcriptional activation domain (Smith et al. 2004), suggesting it may alter protein function.

To determine if the G195E substitution had functional effects, we resurrected the AncYakut and Wrangel Island *NEUROG3* genes and tested their ability to transactivate luciferase expression from a reporter vector containing a minimal promoter and six repeats of the *PAX4* E-box (pGL3[*luc/6x-PAX4E/minP*]) in transiently transfected elephant dermal fibroblasts (fig. 4*C*). The Wrangel Island NEUROG3 transactivated luciferase expression from the pGL3[*luc/6x-PAX4E/minP*] reporter vector more strongly than the AncYakut NEUROG3 protein (1.8-fold, Wilcox *P *=* *0.007), indicating that the NEUROG3 G195E substitution is a hypermorphic mutation. Loss of function mutations in the human *NEUROG3* gene causes congenital malabsorptive diarrhea (DIAR4 [MIM: 610370]), a disorder characterized by neonatal diabetes, chronic unremitting malabsorptive diarrhea, vomiting, dehydration, and severe hyperchloremic metabolic acidosis (Wang et al. 2006; Pinney et al. 2011; Rubio-Cabezas et al. 2011). *NEUROG3* knockout mice die postnatally from diabetes (Rubio-Cabezas et al. 2011) suggesting that the NEUROG3 G195E substitution may have affected insulin signaling in Wrangel Island mammoths.

**Fig. 5:**
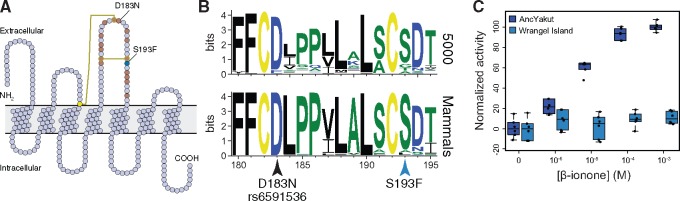
—The Wrangel Island mammoth β-ionone sensor OR5A1 is nonfunctional. (*A*) Snake diagram of the Wrangel Island mammoth OR5A1 protein. The locations of cysteine residues and disulfide bonds are shown as yellow circles and lines, respectively. The locations of residues previously shown by high throughput mutagenesis to affect receptor function are shown as brown circles (Mainland et al. 2014). The locations of the Wrangel Island mammoth S193F substitution and the human D183N polymorphism are also shown. (*B*) Sequence logo showing conservation of OR5A1 AAs 180-195 from 5,000 randomly selected odorant receptors (upper) and mammals (lower). Locations of the S193F substitution and the D183N polymorphism are shown. (*C*) Dose–response curve showing normalized activity of the AncYakut and Wrangel Island mammoth OR5A1 odorant receptors to β-ionone. Data shown are standardized to nontransfected Hana3a cells and no β-ionone, *n *=* *6.

A previous study of the Wrangel Island mammoth genome found a high rate of pseudogenization in olfactory receptors (Rogers and Slatkin 2017), which have greatly expanded in the elephant lineage (Niimura et al. 2014) and generally evolve rapidly through both adaptive and neutral birth–death processes (Nei et al. 2008). Consistent with the importance of an expanded repertoire of olfactory receptors, elephants rely on olfactory cues to locate food and to exclude nonrewarding food options (Plotnik et al. 2014). Similar to Rogers and Slatkin (2017), we found that odorant receptors were the largest class of gene (21/115) with “probably damaging” mutations in the Wrangel Island mammoth genome. Among the olfactory receptors with “probably damaging” amino acid substitutions is *OR5A1*, which encodes the mammalian β-ionone sensor (Jaeger et al. 2013). β-Ionones are of a family of closely related aroma compounds known as rose ketones, which are the major contributor to the aroma of flowers such as roses and violets. Remarkably a human D183N polymorphism (rs6591536), close to the Wrangel Island mammoth OR5A1 S193F (c.578C>T) substitution (fig. 5*A*), underlies differential sensitivity to β-ionone in humans (Jaeger et al. 2013). β-Ionone sensitive individuals, for example, can more easily distinguish food and beverages with added β-ionone than insensitive individuals and typically describe β-ionone as “fragrant” and “floral,” whereas insensitive individuals describe β-ionone as smelling like “sour/acid/vinegar” and “sharp/pungent/acid.”

Both the human D183N and Wrangel Island mammoth OR5A1 S193F substitutions occur in a disulfide bonded extracellular loop that plays a role in ligand recognition (fig. 5*A*) (Man et al. 2004; Zhuang et al. 2009; Mainland et al. 2014; Yu et al. 2015). This site is also nearly invariant for serine in mammalian OR5A1 orthologs as well as 5,000 diverse olfactory receptor paralogs (fig. 5*B*) suggesting that the S193F substitution may affect receptor function. To determine if the S193F substitution had functional consequences, we resurrected the AncYakut and Wrangel Island mammoth *OR5A1* genes and tested their sensitivity to β-ionone using the Hana3A odorant receptor assay (Saito et al. 2004; Zhuang and Matsunami 2008). We found that the AncYakut OR5A1 was strongly activated by β-ionone. In stark contrast, the Wrangel Island mammoth OR5A1 completely lacked β-ionone sensitivity, indicating that the OR5A1 S193F substitution is a loss of function mutation (fig. 5*C*). Forbs were prominent in the diet of Late Quaternary megafauna (Willerslev et al. 2014), including mammoths, suggesting that the S193F substitution in OR5A1 may have altered the ability of the Wrangel Island mammoth to detect one of their major food sources, or that the floral composition of Wrangel Island (Lozhkin et al. 2011) shifted dietary preferences of Wrangel Island mammoths, reducing purifying selection on *OR5A1.*

## Discussion

The final causes of the extinction of Wrangel Island mammoths are mysterious, but it is clear that Wrangel Island mammoths experienced an episode of demographic decline coincident with their isolation leading to a chronically small population. The minimum viable population size (MVPS) to prevent the loss of genetic diversity in wild populations has been estimated to be ∼500, whereas the MVPS to prevent the accumulation of deleterious mutations is ∼1,000 (Thomas 1990; Nunney and Campbell 1993). The adult MVPS for Asian elephants, for example, has been estimated to be 218–266 to 4,700 (Reed et al. 2003; Flather et al. 2011). The effective population size of Wrangel Island mammoths has been estimated to be ∼300–500 (Nyström et al. 2010, 2012; Palkopoulou et al. 2015), close to the 150–800 individual carrying capacity of the island (Nyström et al. 2012). These data suggest that the Wrangel Island mammoth population was at the lower end of its MVPS and too small to effectively purge deleterious mutations. Consistent with expectations for small populations, previous studies of Wrangel Island mammoths have found signatures of reduced genetic diversity, recurrent breeding among distant relatives, and the fixation of putatively deleterious alleles (Nyström et al. 2010, 2012; Thomas 2012; Palkopoulou et al. 2015; Pečnerová et al. 2016; Rogers and Slatkin 2017). These data suggest that deleterious mutations accumulated in Wrangel Island mammoths in response to long-term low effective population size and may have contributed to their extinction (Palkopoulou et al. 2015; Rogers and Slatkin 2017).

We found that the Wrangel Island mammoth genome had numerous homozygous substitutions that are predicted to be deleterious and leading to a unique set of abnormal phenotypes compared with older, continental populations of mammoths, and present day Asian elephants. Consistent with our computational analyses of putatively deleterious substitutions, we validated gain or loss of function mutations in *HYLS1*, *NKD1*, *NEUROG3*, and *OR5A1*, confirming that at least some predicted deleterious mutations were indeed function altering. The loss of function mutation in OR5A1, for example, likely altered the ability of Wrangel Island mammoths to detect β-ionone and thus floral scents, whereas the loss of function mutation in NKD1 may have affected male fertility in Wrangel Island mammoths. The loss of function mutation in HYLS1 may have had more global effects given the widespread importance of cilia in vertebrate development (Badano et al. 2006). In contrast to the other genes we tested, the Wrangel Island specific mutation in *NEUROG3* is a hypermorph rather than a loss of function suggesting it may have caused gain of function phenotypes related to the development and function of pancreatic beta cells (Smith et al. 2004).

Unfortunately, although mammoths are an excellent case study for the evolution of derived phenotypes (Lynch et al. 2015) and the genomics of isolation and extinction (Palkopoulou et al. 2015; Rogers and Slatkin 2017), we are unable to do the kinds of forward and reverse genetic experiments that generally establish causal associations between genotypes and phenotypes. Thus, this study has obvious limitations. We infer, for example, the functional consequences of amino acid substitutions using a computational model that compares the properties amino acids and the likelihood of observing the derived substitution given the pattern of amino acid variation at that site in orthologous genes (Adzhubei et al. 2010, 2013). We cannot know, however, whether the potentially deleterious effects of amino acid substitutions are buffered through epistasis and suppressed or have variable penetrance. Similarly, we infer the phenotypic consequences of deleterious amino acid variants by reference to mouse knockout and human disease data, assuming that the same gene has the same role across species. Although this is often the case, development is plastic and the gene regulatory, morphogenetic, and structural bases of homologous characters can diverge through a process of developmental systems drift (True and Haag 2001; Liao and Zhang 2008; Lynch 2009; Wang and Sommer 2011). Even with these limitations, our computational inferences and functional validation provide experimental evidence that Wrangel Island mammoths may have suffered adverse consequences from their prolonged isolation and small population size. A larger sample size is necessary, however, to determine if the population of mammoths on Wrangel Island had a higher burden of deleterious mutations than other mammoth populations.

## Supplementary Material

evz279_Supplementary_Data
